# Evaluation of pyroptosis-associated genes in endometrial cancer utilizing a 101-combination machine learning framework and multi-omics data

**DOI:** 10.3389/fmed.2025.1590405

**Published:** 2025-06-05

**Authors:** Li Juan Huang, Chen Liu, Lin Chen, Min Tang, Shi Tong Zhan, Feng Chen, An Yi Teng, Li Na Zhou, Wei Lin Sang, Ye Yang

**Affiliations:** ^1^Department of Obstetrics and Gynecology, Shanghai General Hospital, Shanghai Jiao Tong University School of Medicine, Shanghai, China; ^2^Obstetrics and Gynecology Hospital of Fudan University (Shanghai Red House Ob and Gyn Hospital), Shanghai, China; ^3^Department of Surgery, Shanghai General Hospital, Shanghai Jiao Tong University School of Medicine, Shanghai, China; ^4^Department of Obstetrics and Gynecology, Shanghai Songjiang District Maternal and Child Health Care Hospital, Shanghai, China; ^5^Department of Orthopedics, Shanghai General Hospital, Shanghai Jiao Tong University School of Medicine, Shanghai, China

**Keywords:** endometrial cancer, pyroptosis, prognostic model, immune microenvironment, drug sensitivity

## Abstract

**Background:**

Endometrial cancer (EC) is a common and increasingly prevalent gynecological malignancy. Pyroptosis, a pro-inflammatory form of programmed cell death, plays dual roles in cancer but remains poorly understood in the context of EC and its immune microenvironment.

**Methods:**

We identified pyroptosis-associated genes (PAGs) and applied a 101-combination machine learning framework to construct and validate a robust prognostic model using TCGA bulk RNA-seq and single-cell transcriptomic data. Immune infiltration was assessed using CIBERSORT and Tumor Immune Dysfunction and Exclusion (TIDE), while CellChat was employed to investigate pyroptosis-related cell–cell communication. Drug sensitivity was predicted with OncoPredict.

**Results:**

A seven-gene prognostic model demonstrated robust predictive performance with concordance index (*C*-index) values exceeding 0.70 in both training and validation cohorts. The model stratified EC patients into high- and low-risk groups with distinct immune infiltration profiles and differential responses to programmed cell death protein 1 (PD-1) blockade. Drug sensitivity analysis revealed several therapeutic agents with potential efficacy in high-risk and low-risk subgroups.

**Conclusion:**

This study highlights the clinical and immunological relevance of pyroptosis in EC and introduces a PAG-based model with strong predictive and therapeutic potential. These findings provide a foundation for developing pyroptosis-guided precision immunotherapy strategies in EC.

## Introduction

Endometrial cancer (EC) is the second most commonly diagnosed gynecological malignancy worldwide, with a steadily rising incidence, particularly in developed countries. Epidemiological projections from the American Cancer Society estimate that in 2025, EC will contribute to approximately 69,120 newly diagnosed cases and 13,860 related mortalities (1). While early-stage EC has a five-year survival rate of about 95%, advanced-stage disease has a poor prognosis, with survival dropping to 14% (2). These statistics highlight the critical importance of early diagnosis in improving patient outcomes.

Adjuvant cancer therapies have been shown to exert anti-tumor effects, in part, through the induction of pyroptosis (3). This form of cell death is characterized by cellular swelling, osmotic lysis, disruption of membrane integrity, and alterations in electrochemical gradients such as calcium ion (Ca^2+^) flux. These changes result in the release of inflammatory cytokines, including interleukin-1β (IL-1β) and IL-18 (4, 5), which amplify inflammatory responses and facilitate the activation of antigen-specific cytotoxic T lymphocytes (CTLs). These CTLs play a crucial role in recognizing and eliminating tumor cells, initiating the first step of the “tumor-immunity” cycle and suppressing tumor progression (6–10).

Pyroptosis plays a complex and context-dependent role in cancer (8, 11). It can promote tumor growth and metastasis through inflammation and immune evasion (7). The shift from apoptosis to pyroptosis mediated by PD-L1 can promote tumor necrosis (12), potentially facilitating tumor growth and hindering antitumor immunity (13). Gao et al. (14) found that higher GSDMD expression might contribute to tumor evasion of innate immune responses and is associated with poor prognosis in non-small cell lung cancer. On the other hand, pyroptosis also activates antitumor immunity by recruiting immune cells and enhancing immunotherapy response (11, 15, 16). A study demonstrated that GSDME acts as a tumor suppressor by activating pyroptosis and enhancing antitumor immunity (17). Pyroptosis-induced inflammation within the tumor microenvironment (TME) can stimulate the immune system by activating immune cells and pathways, thereby improving the efficacy of cancer immunotherapy (18). It is also associated with many adverse effects of cancer therapy, such as cytokine release syndrome in chimeric antigen receptor T (CAR-T) cell therapy (19) or chemotherapy drug damage to normal tissues in chemotherapy (20).

In recent years, our research team has focused on the field of endometrial cancer pyroptosis. Our previously published article confirms that pyroptosis-related protein nucleotide-binding domain (NOD)-like receptor (NLR) family member pyrin-domain-containing protein 3 (NLRP3), caspase-1, and gasdermin D (GSDMD) were overexpressed in endometrial cancer tissue and cells. GSDMD-induced pyroptosis suppressed tumor growth in subcutaneous EC xenografts, revealing its tumor-suppressive role in EC (21). Furthermore, we also demonstrated Charged Multivesicular Body Protein 4B (CHMP4B) and vacuolar protein sorting 4 homolog A (VPS4A) reverse GSDMD-mediated pyroptosis by cell membrane remodeling in EC. GSDMD knockdown reduced PI-positive cells, Ca^2+^ efflux, IL-1β, and LDH release, while CHMP4B and VPS4A depletion enhanced these indicators in EC cells. Membrane perforations decreased with inactivated GSDMD and increased or decreased after CHMP4B and VPS4A depletion or overexpression in EC cells, suggesting a regulatory mechanism within the pyroptosis pathway in EC (22). Despite these findings, the role and mechanisms of pyroptosis in EC remain incompletely understood.

Understanding these processes would offer new insights and strategies for improving treatment for endometrium cancer (23, 24). Therefore, in this study, we continued to investigate the prognostic significance and regulatory functions of pyroptosis-related genes in endometrial cancer. Using publicly available datasets, we identified key pyroptotic genes, assessed their diagnostic relevance, and explored their associations with the immune microenvironment. Pyroptosis-associated essential genes were identified by the DepMap Public 23Q2 dataset and their involvement in membrane repair and pyroptosis was validated by drug treatment and western blotting analysis in AN3CA and HEC1A cells. This work provides a theoretical basis for future studies and potential therapeutic strategies in EC.

## Method

### Data collection

RNA sequencing (RNA-seq) data were obtained from 589 patients in the TCGA-UCEC cohort (accessed via https://portal.gdc.cancer.gov on August 11, 2024). Due to missing survival data for 17 patients, these cases were excluded from the study. Consequently, our final analysis included mRNA expression data from 538 tumor samples and 34 normal tissue samples, along with survival outcomes and clinical follow-up data corresponding to these patients. Somatic mutation data, provided in mutation annotation format (MAF), were downloaded from TCGA, while copy number variation (CNV) data for TCGA-UCEC patients were retrieved from the UCSC Xena database (accessed via https://xena.ucsc.edu on August 11, 2024).

### Acquisition of pyroptosis-associated genes

A list of 52 pyroptosis-associated genes (PAGs) was retrieved from the MSigDB database (25) (accessed via https://www.gsea-msigdb.org/gsea/msigdb) and selected for further analysis in this study (Supplementary Table 1).

### Analysis of PAGs expression and mutation

In the TCGA-UCEC dataset, the pheatmap package was utilized to generate a heatmap representing the expression of PAGs. Differences in PAGs expression between tumor and normal groups were visualized using boxplots created with the ggplot2 package. For an in-depth examination of the chromosomal distribution of PAGs, the RCircos package was employed to produce circular visualizations. Additionally, mutation data for TCGA-UCEC were downloaded and analyzed using the maftools and oncoplot packages to generate waterfall plots illustrating the mutations present in the selected PAGs.

### Consensus clustering analysis

Consensus clustering is a robust resampling-based technique employed to determine subgroup memberships and validate clustering outcomes. In the present study, we leveraged the ConsensusClusterPlus package (26) in R to categorize distinct pyroptosis subtypes based on the expression profiles of PAGs. The optimal number of clusters (*K*) was determined by evaluating the cumulative distribution function (CDF) plot, delta area plot, and consensus matrix heatmap. Based on these metrics, *K* = 2 was selected as the optimal number of clusters, and samples were accordingly divided into two distinct pyroptosis-related subtypes. The survival disparities among these subtypes were evaluated using Kaplan–Meier (K–M) survival curves, which were generated utilizing the survminer package.

### Screening of PAGs by single-cell RNA sequencing analysis

We acquired single-cell RNA sequencing data from the GSE173682 dataset (27), which presented a comprehensive multi-omic cell atlas of matched single-cell transcriptome and single-cell chromatin accessibility profiles spanning over 150,000 cells from 11 human gynecologic tumors, including samples from five EC patients. To identify the gene expression patterns associated with pyroptosis, single-cell RNA sequencing analysis was conducted from the dataset using the “Seurat” package.

Low-quality cells were removed to eliminate cell-specific biases. Quality control metrics included: cells were retained only if their mitochondrial gene content was below 10%, and genes were included if they were expressed in at least 10 cells within an expression range of 500 to 6,000. Post-quality control, 10,794 cells remained for downstream bioinformatic analyses.

Data normalization and feature selection were performed using the Seurat R package. Gene expression data were normalized using the “LogNormalize” method with a scaling factor of 10,000. Subsequently, the top 2,000 highly variable genes were identified using the “vst” method, which selects genes based on their variance-stabilizing transformation across cells. Principle component analysis (PCA) was performed on the list of highly variable genes and identify cell clusters. We used the elbow method (elbow function in Seurat) to identify significant priniciple components (PCs). The top 20 PCs were used for clustering with the FindClusters function (10 clusters with resolution = 0.5). Marker genes were determined with *p-*value <0.05 and log2(fold-change) >1 by performing differential gene expression analysis between the clusters using the likelihood-ratio test. Cell types were annotated based on the CellMarker 2.0 database (28). To mitigate batch effects across the five samples, we applied the Harmony package. We performed umap analysis using the results of PCA with significant PCs as input. The top 20 PCs were used for clustering and 18 subclusters were obtained with resolution = 0.5.

We utilized the AddModuleScore function from the Seurat package to calculate a pyroptosis score for each cell. This function computes the average expression level of PAGs, subtracted by a control gene set matched by expression bins, thus reflecting the relative activation level of the pyroptosis gene module across cells. The resulting Pyroptosis_Score was visualized on a umap embedding using feature plot, and cells were stratified into “high” and “low” groups based on the median score.

To identify genes differentially expressed between the two groups, we performed a differential expression analysis using the FindMarkers function. Genes were considered significantly differentially expressed if they met the criteria of *p* < 0.05 and |avg_log2FC| >1. We then intersected the list of significant DEGs with our predefined PAG list to extract pyroptosis-associated DEGs (PADEGs). Representative genes were visualized using density plots generated with the Nebulosa package to highlight their expression distributions across cell populations.

### Construction and evaluation of the risk model

The TCGA-UCEC dataset was randomly partitioned into a training set and an internal validation set in a 7:3 ratio, ensuring a balanced distribution of clinical characteristics between the two cohorts. We utilized the Mime1 package to apply 10 machine learning algorithms (29), including Lasso, Ridge, stepwise Cox, CoxBoost, random survival forest (RSF), elastic net (Enet), partial least squares regression for Cox (plsRcox), supervised principal components (SuperPC), generalized boosted regression modeling (GBM), and survival support vector machine (survival-SVM). Ten of these models were individual algorithms applied alone, while the remaining models consisted of combinations, including a feature selection algorithm (Lasso, RSF, Boruta) and a survival prediction algorithm (Cox, survival-SVM, GBM, etc.). Variable selection and model development were conducted within the TCGA-UCEC training dataset using a 10-fold cross-validation approach. All constructed models were subsequently validated in the TCGA internal validation set.

A univariate Cox proportional hazards regression analysis was performed on all candidate genes to filter out irrelevant features and genes with a *p*-value less than the specified cutoff (unicox_p_cutoff = 0.05) were retained for downstream analysis. We choosed the “all” mode in the ML.Dev.Prog.Sig function of Mime1 package to explore both single-gene and multi-gene interactions to identify the most predictive signature. The nodesize = 5 parameter defines the minimum number of samples needed for a node split in tree-based modeling to ensure robustness against overfitting.

The concordance index (*C*-index) was calculated for both the training and internal validation sets to evaluate predictive performance. Besides, 1-year-AUC was also calculated to assist in the selection of the final model. The most robust and clinically relevant algorithm combination (RSF) was selected for further investigation. We employed the RSF algorithm implemented in the random Forest SRC package to develop a prognostic model using the training dataset. To optimize model performance, we performed hyperparameter tuning using the tune function. The optimal parameter combination was determined based on out-of-bag (OOB) error rates. The final RSF model was then trained with the identified optimal parameters (ntree = 1,000, mtry = 28, nodesize = 3).

To evaluate the model’s ability to predict survival probabilities at specific time points (1-year, 2-year, and 3-year survival), we constructed time-dependent ROC curves using the timeROC package. Risk scores were generated for both the training and validation datasets using the trained RSF model. Patients were stratified into high-risk and low-risk groups based on the median risk score derived from the training set. Kaplan–Meier survival curves were plotted for both groups, and log-rank tests were performed to assess the statistical significance of differences in survival probabilities.

The importance of individual genes in predicting survival outcomes was quantified using the variable importance metric (VIMP) derived from the RSF model. The top-ranked genes were identified based on their VIMP scores and visualized using bar plots. These genes were further used as input features for downstream Cox proportional hazards regression analysis.

A nomogram was constructed via the rms package to predict patient survival probabilities at specific time points (2-year, 3-year, and 5-year survival). A multivariate Cox proportional hazards model was fitted using the top-ranked genes identified from the RSF analysis. Calibration curves were plotted to assess the agreement between predicted and observed survival probabilities.

### Immune infiltration and immunotherapy analysis

We utilized the CIBERSORT (30) package in R to quantify the proportions of 22 immune cell types in tumor samples, including seven T cell subtypes, three B cell subtypes, NK cells, and myeloid cells. The results were saved for subsequent analysis.

To provide effective guidance for tumor immunotherapy, we obtained immune phenotype scores (IPS) from The Cancer Immunome Atlas (TCIA) (accessible at https://tcia.at/) (31). These scores were utilized to predict responses to immune checkpoint blockade in the training cohort. Wilcoxon rank-sum tests were employed to evaluate the disparities in responses to cytotoxic T lymphocyte antigen-4 (CTLA-4) inhibitors and anti-PD-1/PD-L1 inhibitors across different risk strata.

### PAGS and immunotherapy for endometrial cancer

GSE251923 is a dataset which indicates responders and non-responders to anti-PD-1 therapy in human EC (32). The clustering, visualization, and annotation methods for this dataset are as described above. Cell–cell interaction analysis was performed using the CellChat package (version 1.6.0) (9). Signaling pathway networks were evaluated using this package.

### Drug sensitivity analysis

OncoPredict is a computational tool tailored for predicting the responsiveness of tumor patients to a range of chemotherapy and targeted therapeutic agents (33). Leveraging patient gene expression profiles and established drug sensitivity data, it generates predictive models. In the present study, patients were stratified into distinct expression clusters (PagCluster) based on the median expression levels of prognostic genes. Subsequently, the drug sensitivity of these clusters to commonly used chemotherapy drugs was evaluated.

### Identification of pyroptosis-associated essential genes

The dependency scores (Chronos) of 27 endometrial cancer cell lines were obtained from the DepMap Public 23Q2 dataset (accessed on February 24, 2024). Genes with gene effect scores <−1 were selected as potential pyroptosis-associated essential genes. Candidate genes involved in membrane repair and pyroptosis were prioritized for further validation based on biological relevance.

### Cell culture and drug treatment

AN3CA cells and HEC1A cells were obtained from the American Type Culture Collection. They were cultured in modified Eagle medium (MEM) (Servicebio, China) with 10% FBS (Vazyme, China). The cells were cultured at 37°C in a humidified incubator with 5% CO_2_.

Cells were treated with LPS (Sigma-Aldrich, United States; 50 ng/mL, 4 h) and nigericin (Sigma-Aldrich, United States; 10 μM, 30 min); LPS, nigericin and olaparib (Beyotime, China; 200 nM, 24 h); LPS, nigericin and niraparib (Beyotime, China; 50 nM, 24 h); LPS, nigericin and EDTA (Absin, China; 1 mM, 12 h); LPS, nigericin and CaCl₂ (Macklin, China; 100 μM, 12 h); vehicle (DMSO) at the same volume.

After drug treatments, cells were harvested for downstream assays by western blotting.

### Coimmunoprecipitation assays

Coimmunoprecipitation (Co-IP) assays were performed as described previously. The cells were lysed with RIPA lysis buffer (Epizyme, China) supplemented with protease and phosphatase inhibitors (Epizyme, China). The lysate was sonicated for 30 s and kept on ice for 1 h, and the supernatant was collected. Mixed 50 μL protein A/G magnetic beads (Beyotime Biotechnology, China) into the protein-antibody solution and incubated on a shaker at 4°C for 3 h. The magnetic beads were precipitated with a magnetic stand. The magnetic beads were boiled with SDS loading buffer and subjected to immunoblotting analysis.

### Western blotting

Western blot analysis was used to assess the expression levels of GSDMD, cleaved-GSDMD, CHMP4B, TSG101, PARP1, VPS4A. GAPDH was used as a loading control.

Total proteins were extracted from cultured cells using RIPA lysis buffer (Epizyme, China) supplemented with protease and phosphatase inhibitors (Epizyme, China). Protein concentrations were measured using a BCA Protein Assay Kit (Epizyme, China) according to the manufacturer’s instructions. Equal amounts of protein (30 μg) were resolved by 10% SDS-PAGE and transferred onto PVDF membranes (Vazyme, China). Membranes were blocked with 5% non-fat milk in TBST for 1 h at room temperature and incubated overnight at 4°C with primary antibodies against GSDMD (1:1,000, Proteintech), CHMP4B (1:1,000, Proteintech), TSG101 (1:1,000, Proteintech), VPS4A (1:1,000, Abcam), PARP1 (1:1,000, Abcam) or GAPDH (1:5,000, Proteintech) as a loading control. After washing, membranes were incubated with HRP-conjugated secondary antibodies (1:5,000, Servicebio, China) for 1 h at room temperature. Immunoreactive bands were detected using enhanced chemiluminescence (ECL) reagents (Epizyme, China) and visualized with a ChemiDoc Imaging System (Bio-Rad, United States).

## Result

### Expression and mutation analysis of PAGs

**Figure 1:**
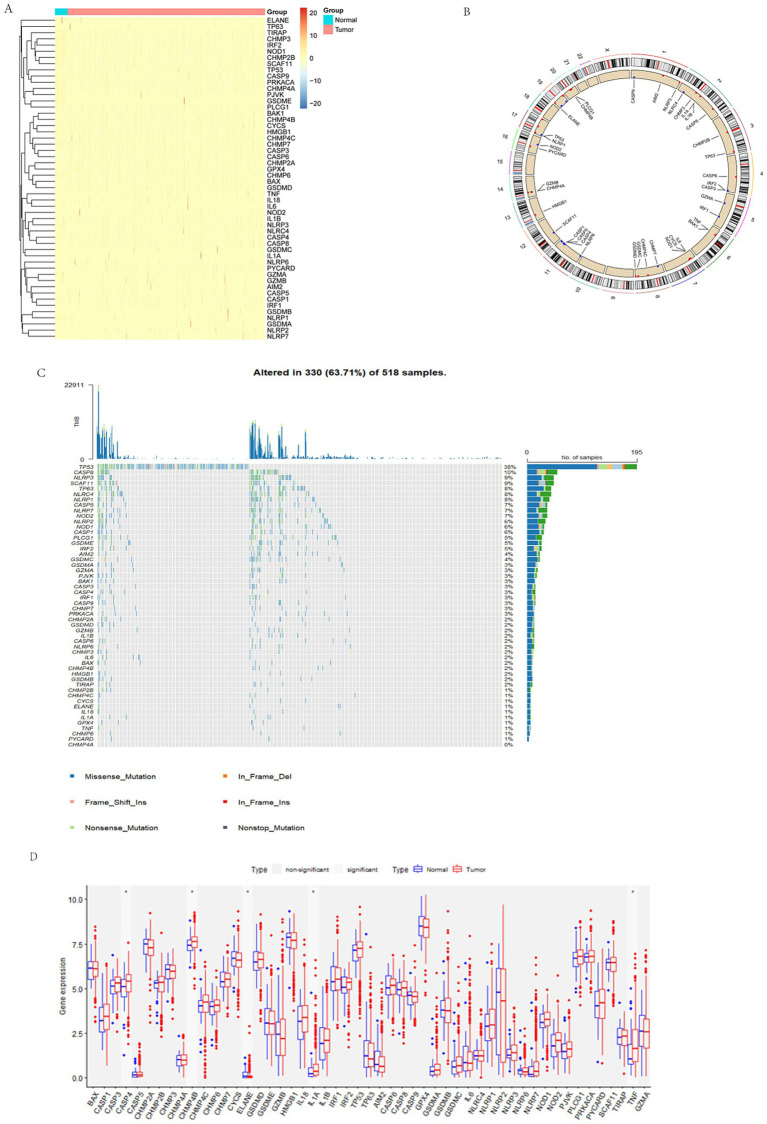
Overview of PAGs in endometrial cancer. **(A)** Heatmap of PAGs expression. The blue bar above represents tumors, while the red bar represents normal tissues. PAGs exhibit medium to high expression in both normal endometrium and endometrial cancer. **(B)** Chromosomal distribution of PAGs. **(C)** Mutation landscape of PAGs in endometrial cancer. TMB distribution plot illustrating the genetic alterations in 518 samples, with 330 (63.71%) showing mutated genes. The x-axis represents different gene names, while the y-axis indicates the number of alterations for each gene. The bar chart, with various colors, depicts different types of mutations, including missense mutations, frameshift insertions/deletions, nonsense mutations, etc. The percentage bar chart on the right displays the proportion of samples with each type of mutation. Color code: blue: missense mutations; orange: frameshift insertions/deletions; pink: frameshift substitutions; red: non-frameshift insertions; green: nonsense mutations; gray: non-stop codon mutationsormal tissues. **(D)** Differential expression of PAGs between tumor and normal tissues. Red represents tumor tissue, and blue represents non-tumor tissue; CASP4, CHMP4B, IL-1A, and TNF are significantly overexpressed in tumors, while ELANE is more highly expressed in normal tissues.

In the TCGA-UCEC dataset, we utilized the “pheatmap” package to construct a heatmap depicting the expression profiles of pyroptosis-associated genes (PAGs), providing a comprehensive visualization of their transcriptional patterns (Figure 1A). To illustrate the chromosomal distribution of PAGs, we employed the “RCircos” package, revealing their widespread localization across autosomes, with notable absences on chromosomes 9, 10, 15, 18, 21, and 22 (Figure 1B).

To investigate the mutational landscape of PAGs in the 338 patients with available data, we applied the “maftools” and “oncoplot” packages to generate waterfall plots, identifying TP53, CASP8, and NLRP3 as the most frequently mutated genes, each exhibiting a mutation rate exceeding 10%. Among the observed alterations, missense mutations were the most prevalent, followed by nonsense mutations (Figure 1C). Furthermore, we utilized the ggplot2 package to generate boxplots comparing PAG expression levels between tumor and normal tissues. Differential expression analysis revealed that CASP4, CHMP4B, ELANE, IL-1A, and TNF were significantly upregulated in tumor samples (*p* < 0.05) (Figure 1D). These findings underscore the transcriptional and mutational heterogeneity of PAGs in endometrial cancer, providing insights into their potential roles in tumorigenesis and immune regulation.

### Consensus clustering analysis for EC classification

**Figure 2:**
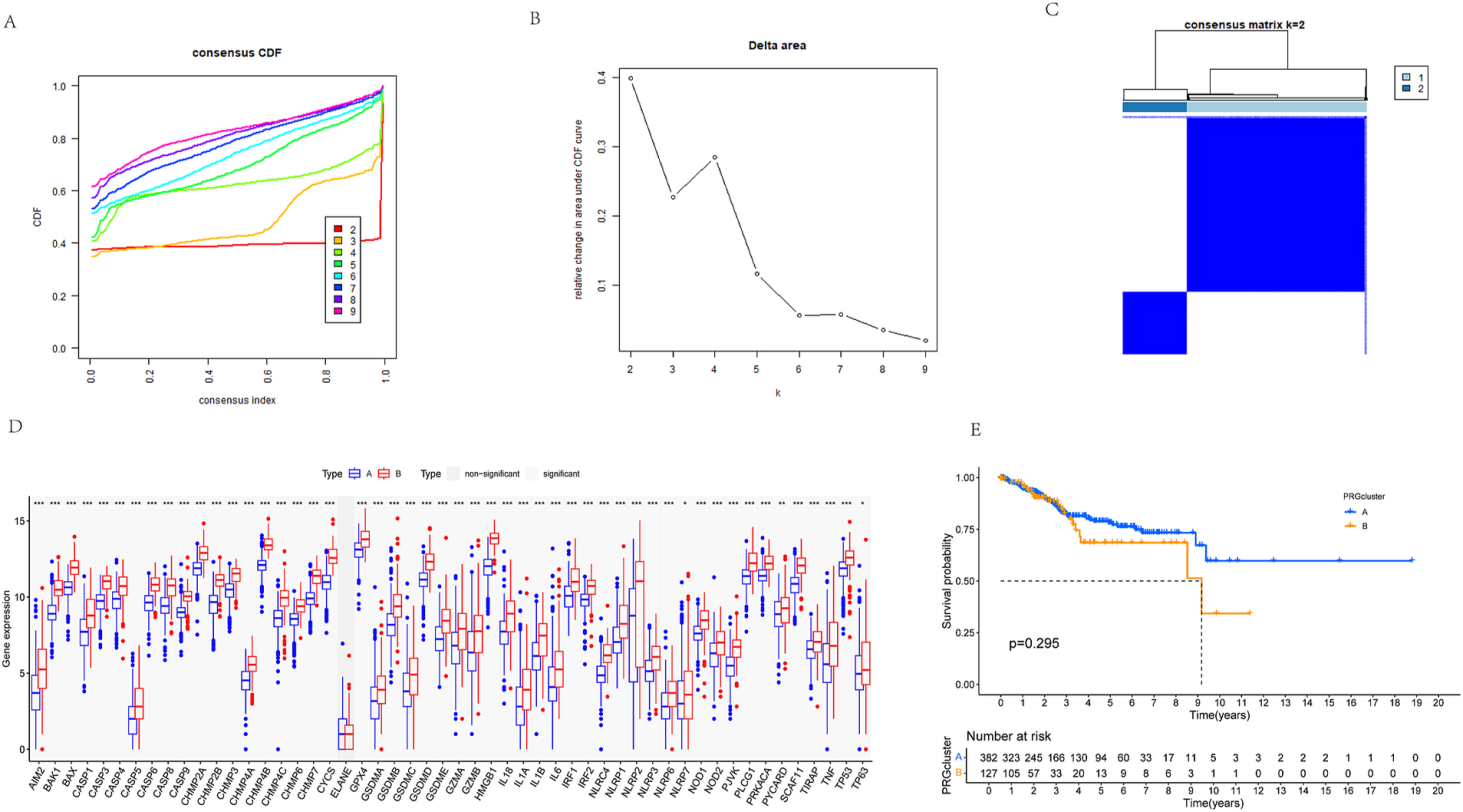
Consensus clustering analysis of TCGA-UCEC tumor samples. **(A)** Cumulative distribution function (CDF) plot of consensus clustering. It describes the changing trends of the CDF of the consensus index under different *K* values. The colors from red to pink represent *K* values ranging from 2 to 9. As *K* increases, the consensus index shows noticeable changes. **(B)** Delta area plot. The x-axis represents the *K* values, while the y-axis shows the relative delta area. As *K* increases, the delta area gradually decreases. **(C)** A consensus matrix heatmap is shown for *K* = 2. **(D)** Differential gene expression between different pagClusters. The x-axis represents the names of different genes, and the y-axis represents the expression level of the gene. The blue box represents the expression level of cluster A, and the red box represents the expression level of cluster B. **(E)** Kaplan–Meier survival analysis of different pagClusters. Cluster A shows a better survival rate in the long term.

Consensus clustering is a widely utilized approach in cancer classification, enabling the identification of molecular subtypes based on gene expression patterns. In this study, we performed consensus clustering on tumor samples from the TCGA-UCEC dataset using the Consensus Cluster Plus package, with classification based on the expression profiles of PAGs. The robustness of the clustering results was assessed through the cumulative distribution function (CDF) plot, delta area plot, and consensus matrix heatmap (Figures 2A–C). Our analysis identified an optimal clustering solution at *K* = 2, stratifying the cohort into two distinct molecular subtypes, designated pagCluster A and pagCluster B.

Differential gene expression analysis between the two subtypes revealed significantly higher expression levels of PAGs in pagCluster B compared to pagCluster A (*p* < 0.05 for all genes except ELANE) (Figure 2D). Based on these expression patterns, pagCluster A was classified as the low pyroptosis expression group, whereas pagCluster B represented the high pyroptosis expression group.

To investigate the prognostic implications of these molecular subtypes, we performed Kaplan–Meier (K-M) survival analysis using the survminer package (Figure 2E). While both groups exhibited relatively high survival rates within the first 3 years, a divergence in survival outcomes became evident over time. Although the difference did not reach statistical significance (*p* = 0.295), patients in pagCluster A consistently demonstrated a slightly higher survival rate than those in pagCluster B across the entire follow-up period. Notably, this survival advantage became more pronounced beyond the 9-year mark, suggesting a potential association between lower PAG expression and improved long-term prognosis. These findings suggest that heightened pyroptosis-related gene expression may be linked to unfavorable clinical outcomes in endometrial cancer, underscoring the potential impact of pyroptosis on disease progression and patient survival. Further investigations are warranted to elucidate the mechanistic basis of this relationship and its implications for therapeutic strategies.

### Pyroptosis scores of PAGs at the single-cell level

To elucidate the gene expression patterns associated with pyroptosis at single-cell resolution, we performed single-cell RNA sequencing (scRNA-seq) analysis using data from the GSE173682 dataset.

**Figure 3:**
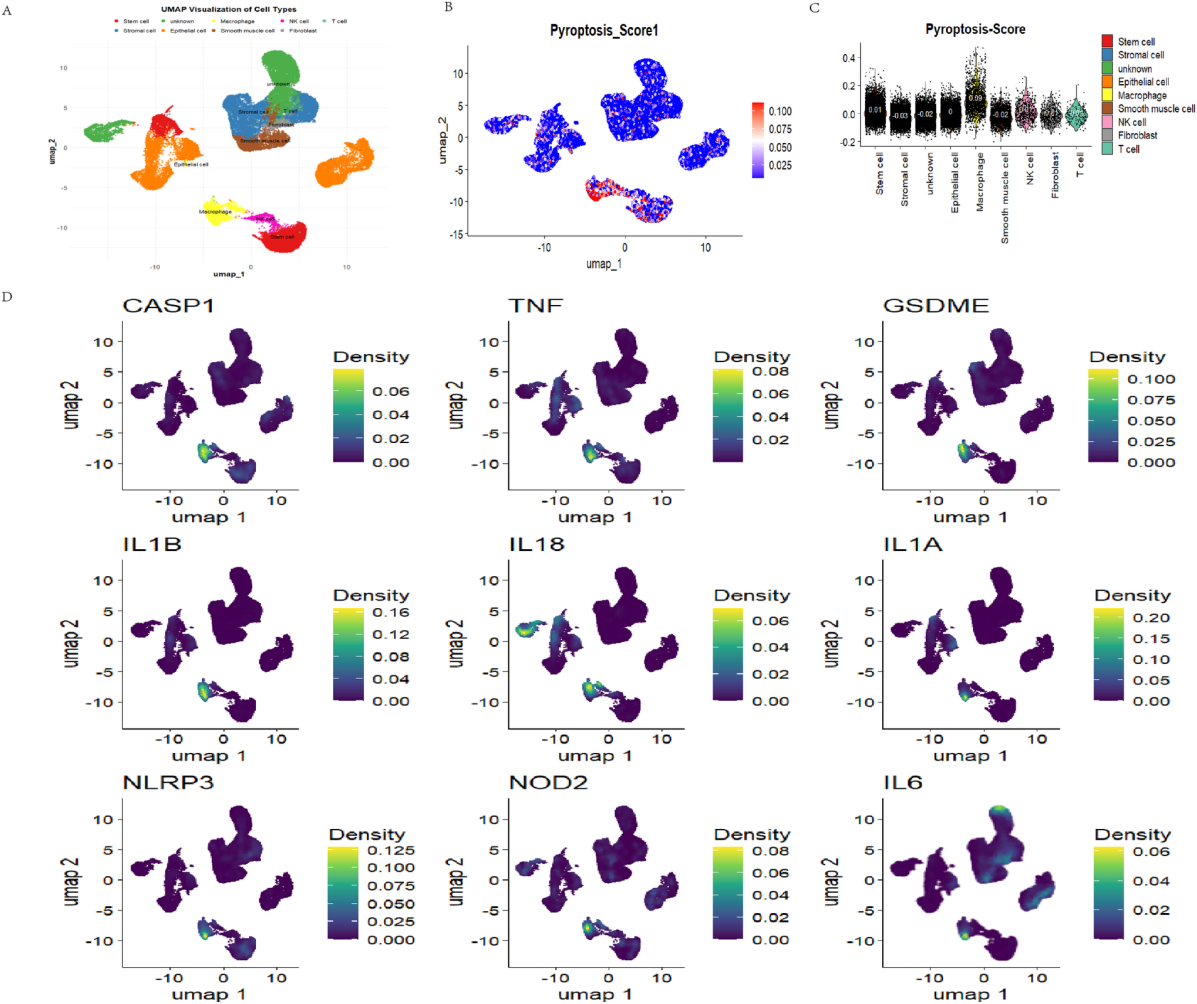
PAGs scoring and distribution in endometrial cancer single cells. **(A)** Clustering and annotation of single cells in endometrial cancer. **(B,C)** PAGs activity scores across single cells. PAGs were scored by “AddModuleScore” function **(B)** and visualized using violin plots **(C)**. Macrophages exhibit a high PAG score. **(D)** Expression distribution of PADEGs2. CASP1, TNF, GSDME, IL1B, IL18, IL1A, NLRP3, NOD2, and IL6 showed aggregation in macrophages.

Following clustering and cell-type annotation, a total of 18 distinct clusters were identified, corresponding to nine major cell subpopulations: stem cells, stromal cells, epithelial cells, smooth muscle cells, fibroblasts, macrophages, NK cells, T cells, and an “unknown” group (Figure 3A).

We applied the “AddModuleScore” function in Seurat. The analysis revealed that PAG activity varied significantly across different cell types, with the highest scores observed in macrophages (0.09) and the lowest in stromal cells (−0.03) (Figures 3B–C).

To further delineate the molecular features associated with pyroptosis, Wilcoxon rank-sum tests were employed to identify differentially expressed genes (DEGs) between high and low PAG score groups (*p* < 0.05), yielding a set of pyroptosis-associated differentially expressed genes (PADEGs) (Table 1). UMAP visualization of PADEGs revealed that key pyroptosis-related genes, including CASP1, TNF, GSDME, IL1A, NLRP3, IL6, NOD2, IL18, and IL1B, were predominantly enriched in macrophages exhibiting high PAG activity (Figure 3D).

**Table 1:** PADEGs in single cell.

Gene	p_val	avg_log2FC	pct.1	pct.2	p_val_adj
CHMP4A	0	1.37534716	0.405	0.205	0
BAX	6.143 × 10^−288^	1.08899767	0.443	0.268	1.37 × 10^−283^
IL1B	8.815 × 10^−283^	6.54089959	0.135	0.03	1.966 × 10^−278^
GSDMD	1.206 × 10^−241^	1.34656643	0.258	0.12	2.689 × 10^−237^
CASP8	9.941 × 10^−223^	1.60427271	0.219	0.095	2.217 × 10^−218^
CASP4	1.425 × 10^−202^	1.14851609	0.323	0.185	3.177 × 10^−198^
CASP1	3.62 × 10^−199^	2.39127742	0.12	0.034	8.072 × 10^−195^
IL18	8.077 × 10^−184^	2.40895224	0.102	0.026	1.801 × 10^−179^
PYCARD	1.154 × 10^−136^	1.40846291	0.172	0.083	2.573 × 10^−132^
TNF	7.81 × 10^−122^	3.00916778	0.079	0.024	1.742 × 10^−117^
IL1A	2.381 × 10^−117^	4.43685994	0.05	0.008	5.308 × 10^−113^
CASP6	8.803 × 10^−107^	1.25646108	0.127	0.059	1.963 × 10^−102^
GZMB	1.798 × 10^−106^	3.37715548	0.052	0.011	4.01 × 10^−102^
CHMP4C	1.4081 × 10^−83^	1.21074597	0.106	0.05	3.1397 × 10^−79^
CHMP6	2.2612 × 10^−74^	1.10457859	0.118	0.062	5.0419 × 10^−70^
PRKACA	3.1219 × 10^−73^	1.12379997	0.112	0.057	6.9611 × 10^−69^
TP53	1.6038 × 10^−67^	1.0309549	0.119	0.065	3.576 × 10^−63^
GSDME	2.3472 × 10^−67^	1.57602404	0.059	0.022	5.2337 × 10^−63^
IL6	3.6123 × 10^−62^	1.98527293	0.124	0.072	8.0547 × 10^−58^
GZMA	4.5224 × 10^−61^	3.50867058	0.022	0.003	1.0084 × 10^−56^
CASP9	1.3908 × 10^−40^	1.20829617	0.048	0.021	3.1011 × 10^−36^
NLRP2	6.1952 × 10^−39^	1.267805	0.04	0.017	1.3814 × 10^−34^
NLRP3	2.6467 × 10^−34^	1.91118671	0.031	0.012	5.9017 × 10^−30^
NOD2	1.5627 × 10^−24^	2.07572334	0.018	0.006	3.4845 × 10^−20^
NOD1	1.3482 × 10^−19^	1.27444705	0.025	0.012	3.0062 × 10^−15^
GSDMB	8.6712 × 10^−17^	1.19040758	0.027	0.014	1.9335 × 10^−12^
TIRAP	1.796 × 10^−16^	1.57603085	0.02	0.009	4.0046 × 10^−12^

### Construction and evaluation of a risk prediction model

**Figure 4:**
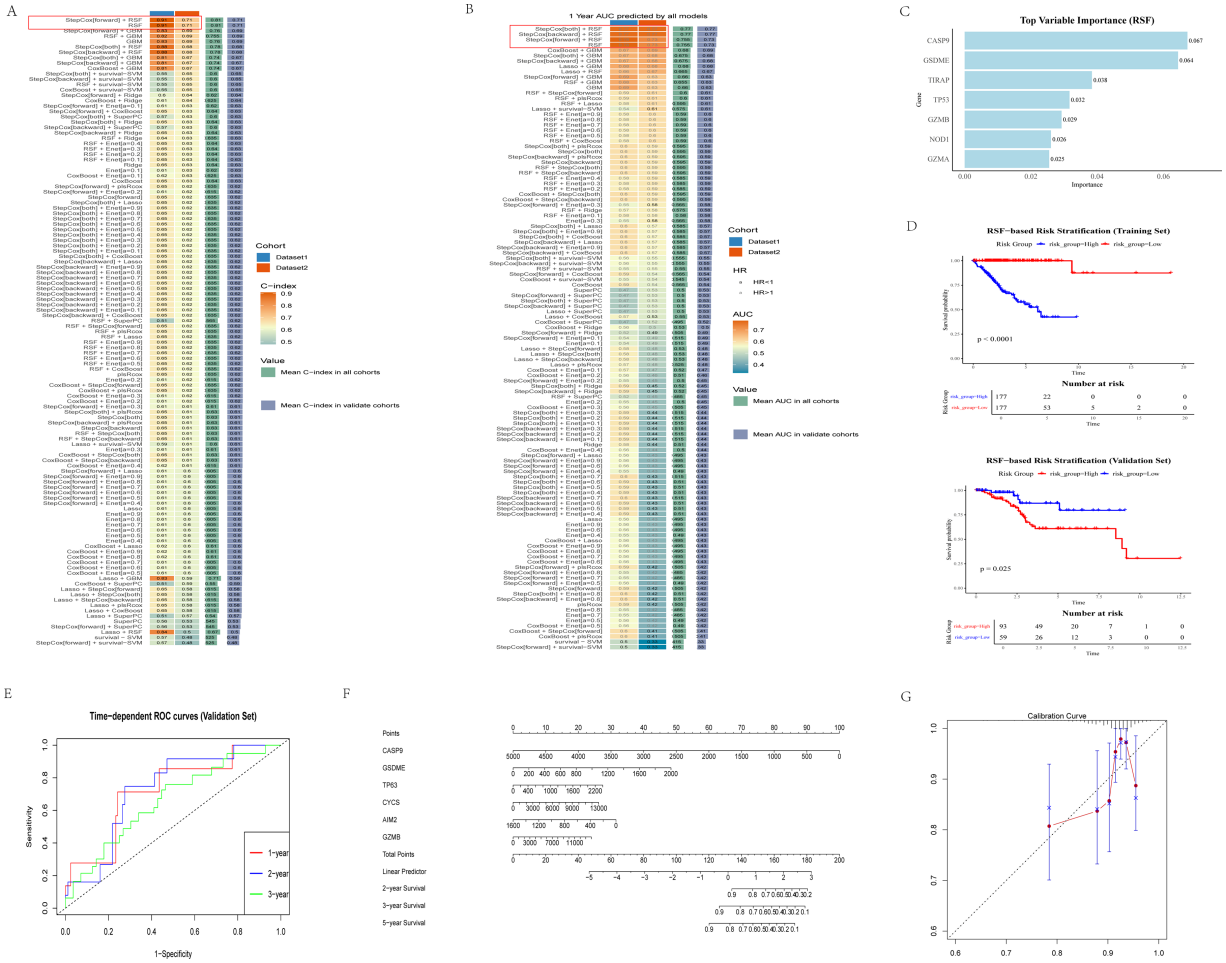
Construction of the risk model. **(A)** *C*-index values of 101 machine learning models. RSF showed the highest *C*-index (0.81 in average, framed in red). **(B)** AUC at 1-year values of 101 machine learning models. RSF showed a high AUC (0.755 in average, framed in red). **(C)** Top variable importance in RSF model for pyroptosis genes. Bar plot showing the importance of pyroptosis-related genes in the RSF model. The x-axis represents the importance score, and the y-axis lists the genes. **(D)** Kaplan–Meier curve of the COX model. Divided by the median, red represents the high-risk group and green represents the low-risk group. The low-risk group enjoys a higher survival rate. **(E)** 1-year, 3-year, and 5-year ROC of the RSF model. (Represented by red, green, and blue respectively). **(F)** Nomogram of a model for estimation of the probability of EC survival. Points were assigned to parameters by drawing lines upward from the corresponding values to the “Points” line. The sum of these points, plotted on the “Total points” line, corresponds to the predicted two, three, and five-year survival. **(G)** Calibration plot of the nomogram. The predictive line (solid line in red) overlaps well with the ideal line (dotted line), indicating that the predictive value approximates the actual value. ^*^p < 0.05, ^**^p < 0.01, and ^***^p < 0.001.

To identify key pyroptosis-associated genes with prognostic significance, we utilized the TCGA-UCEC dataset, randomly dividing 70% of the cohort into a training set and allocating the remaining 30% to a validation set. We constructed multiple predictive models within the training set using the “Mime1” package in R. After rigorous evaluation across both training and validation cohorts, the model integrating Random Survival Forest (RSF) was identified as the optimal approach. This model demonstrated the highest predictive performance, achieving an average *C*-index of 0.81, with C-indices of 0.91 in the training set and 0.71 in the validation set (Figure 4A). Additionally, the one-year survival prediction yielded an area under the curve (AUC) of 0.78 in the training set and 0.73 in the test set, with a hazard ratio (HR) >1, suggesting that elevated expression of pyroptosis-associated genes (PAGs) is correlated with poorer prognosis (Figure 4B).

In the RSF model, the genes with the highest importance scores were CASP9 (0.067), GSDME (0.064), TIRAP (0.038), TP53 (0.032), GZMB (0.029), NOD1 (0.026), and GZMA (0.025). These results indicate that CASP9 and GSDME are the most significant contributors to the predictive power of the RSF model in the context of patient prognosis (Figure 4C). Kaplan–Meier survival analysis based on the median risk score revealed that patients in the low-risk group had significantly better survival outcomes (*p* < 0.0001 in the training cohort; *p* = 0.025 in the testing cohort, Figure 4D).

To further assess the model’s predictive capacity, time-dependent ROC curves were generated for 1-, 2-, and 3-year survival. The results demonstrated robust discriminative ability of the RSF model (Figure 4E). The nomogram was constructed using the *rms* package. Among them, GSDME and several other genes were closely associated with patient survival, which was consistent with the findings from the RSF model (Figure 4F). Finally, calibration curve analysis revealed good agreement between predicted and observed survival probabilities, supporting the strong calibration performance of the nomogram model (Figure 4G).

### Immune checkpoint analysis and immune infiltration analysis

**Figure 5:**
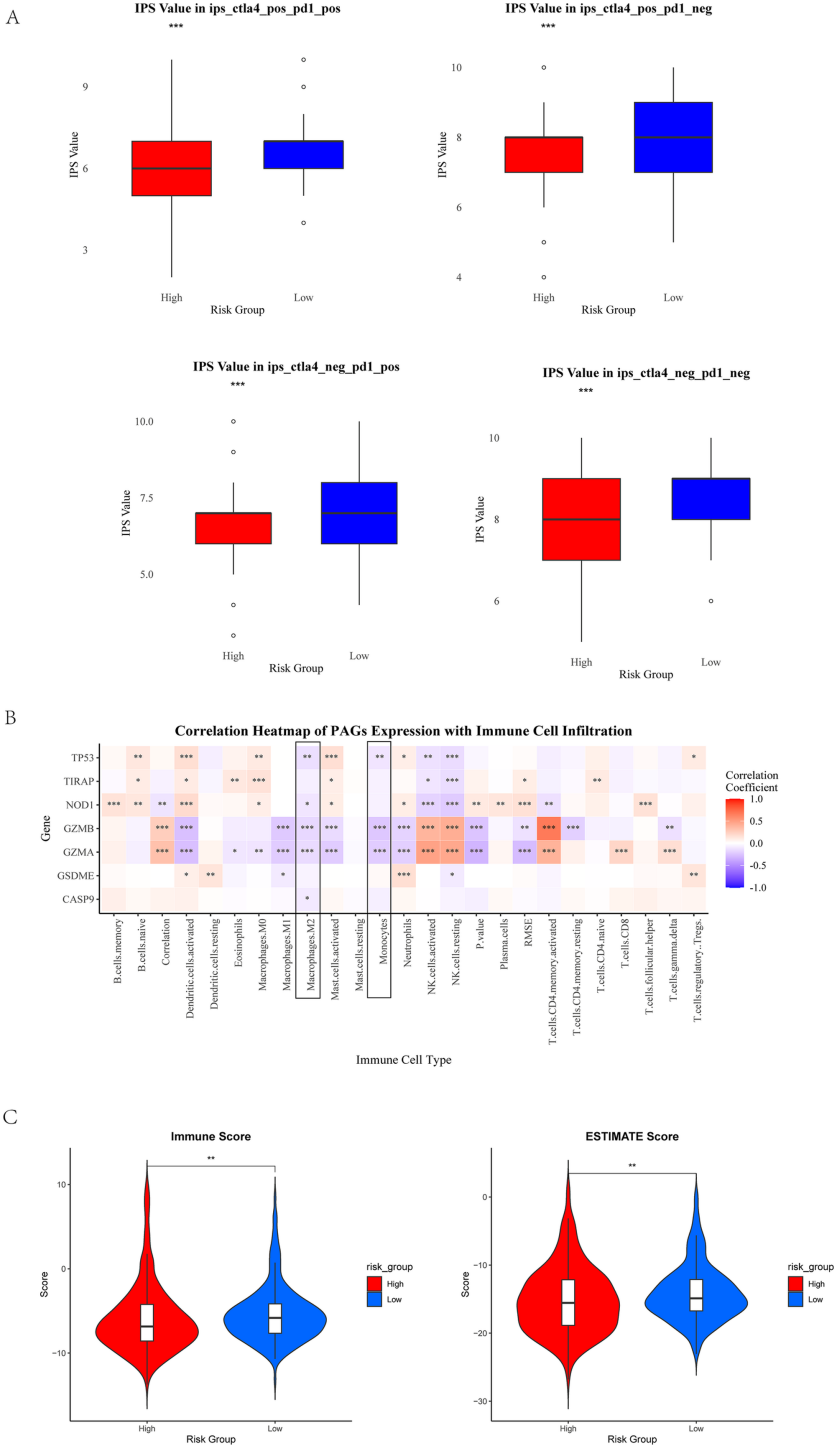
Immune checkpoint analysis and immune infiltration analysis. **(A)** IPS scores in different risk groups. Red represents high-risk group, and blue represents low-risk group. The high-risk group showed a negative predictive result for PD-1 treatment. The high-risk group exhibits a lower IPS. **(B)** The correlation between PAGs and immune cells. Macrophages M2 and monocytes show a negative correlation significantly with PAGs (framed in black). **(C)** Immune score and ESTIMATE score in high-risk group and low-risk group. ^*^p < 0.05, ^**^p < 0.01, and *p* < 0.001.

To elucidate the role of PAG expression in shaping the immune landscape and guiding immunotherapy strategies in EC, we obtained immune phenotype scores (IPS) from The Cancer Immunome Atlas (TCIA) to predict responses to immune checkpoint blockade (ICB) within the training cohort. IPS serves as a predictive biomarker for responsiveness to immune checkpoint inhibitors (ICIs), including PD-1 and CTLA-4 inhibitors. Using the Wilcoxon test, we analyzed differences in IPS between high- and low-risk groups across different immunotherapy modalities. Our results revealed that the effect of immunotherapy under the PAGs signature indicated that patients in the low-risk group had higher immunotherapy scores for four types of immunotherapy, including no CTLA4 and PD1 treatment, PD1 treatment alone, CTLA4 treatment alone, combined PD1 and CTLA4 treatment (*p* < 0.001, Figure 5A).

To further investigate the relationship between PAG expression and immune cell infiltration, we utilized the CIBERSORT algorithm to quantify immune cell composition and assess correlations between PAGs and immune cell subtypes. Our analysis demonstrated a negative correlation between PAG expression and the infiltration of M2 macrophages and monocytes (Figures 5B, *p* < 0.05), suggesting a potential link between pyroptosis-related inflammation and the tumor immune microenvironment (TIME). Additionally, ESTIMATE analyse revealed that patients with low PAG expression exhibited higher immune score (*p* < 0.01) and ESTIMATE score (*p* < 0.01), indicating a more inflamed tumor microenvironment (Figure 5C).

### PAGs in immunotherapy for endometrial cancer

**Figure 6:**
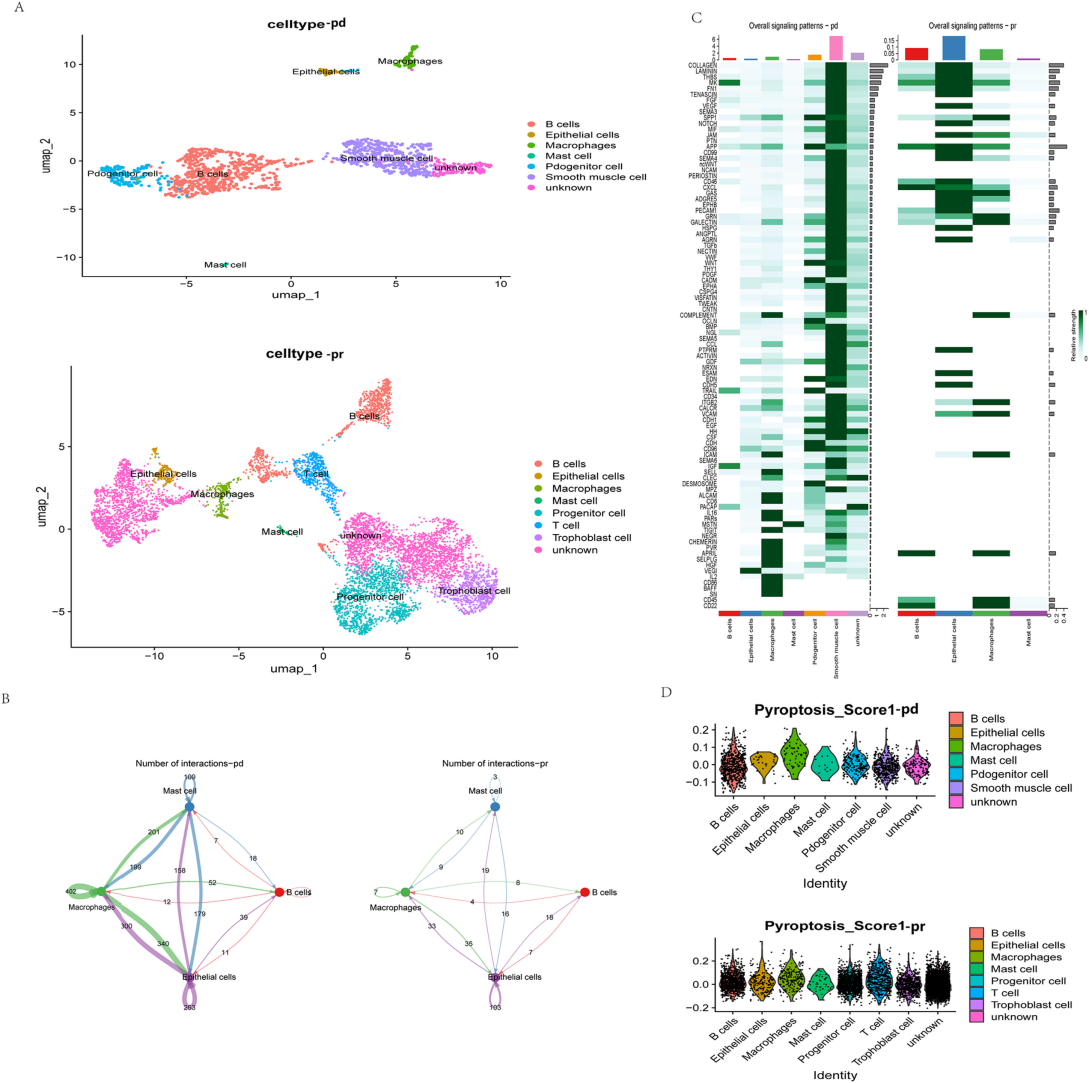
Single-cell analysis of the relationship between anti-PD-1 therapy, immunity, and pyroptosis. **(A)** The umap plot of cell types in the PD and PR group. UMAP visualization results show the distribution of different cell types in two dimensions. The PR group includes B cells, epithelial cells, macrophages, mast cells, progenitor cells, smooth muscle cells, and unknown cells. The PD group contains B cells, epithelial cells, macrophages, mast cells, progenitor cells, T cells, trophoblast cells, and unknown cells. The legend lists the corresponding colors for each cell type. **(B)** Interactions of same cells in the two groups. Blue represents mast cells, green represents macrophages, pink represents B cells, and purple represents epithelial cells. The numbers represent the number of interactions. **(C)** Overall signal patterns of the two groups. **(D)** Pyroptosis scores in each cell type. The score of each cell type is presented in the form of a scatter plot, with the x-axis representing the cell type and the y-axis representing the score value.

To further elucidate the role of PAGs in the context of EC immunotherapy, we analyzed the GSE251923 single-cell dataset, which includes EC patients stratified based on their response to PD-1 blockade therapy. Patients were categorized into two groups: partially responsive (PR) and progressive disease (PD). Following dimensionality reduction using UMAP, we observed that the PR group exhibited increased T cell infiltration compared to the PD group (Figure 6A), suggesting a potential association between T cell activity and response to PD-1 therapy. To further explore cellular interactions in response to PD-1 blockade, we employed the “CellChat” package to construct a cellular communication network among key cell types, including B cells, mast cells, macrophages, and epithelial cells. Notably, in the PR group, macrophage-mediated communication with nearly all other cell types was markedly reduced, implying that PD-1 blockade may modulate macrophage function and intercellular signaling (Figure 6B).

We further examined signaling pathway alterations between the PR and PD groups (Figure 6C). Within macrophages, pathways such as CD56 and BAFF signaling were generally downregulated, suggesting that PD-1 blockade may attenuate pyroptosis-related macrophage activation. Given the previously identified significant role of PAGs in modulating T cell-macrophage interactions, we next performed pyroptosis scoring on cells from both groups using the “AddModuleScore” function in Seurat. Notably, in the PR group, epithelial cells exhibited significantly lower pyroptosis scores, indicating a potential correlation between epithelial cell pyroptosis and response to immunotherapy (Figure 6D). These findings suggest that pyroptosis in epithelial cells may play a pivotal role in determining response to PD-1 blockade in EC patients.

### Drug sensitivity analysis

**Figure 7:**
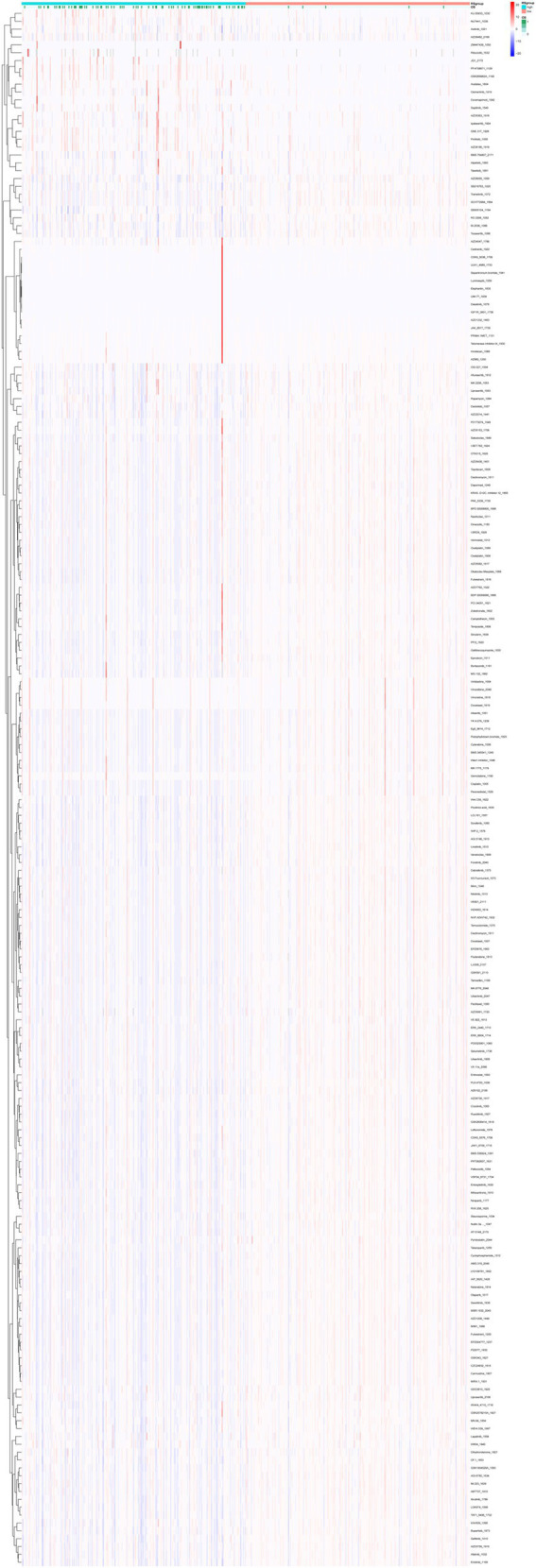
Drug sensitivity analysis in EC. In the RS group, the color scheme differentiates the low-risk cohort with green and the high-risk cohort with red. The transition in IC50 values from low to high is depicted through a gradient ranging from blue to red. The visualization clearly indicates that the IC50 for the low-risk group is inferior to that of the high-risk group, suggesting that a lower expression of PAGs may confer increased sensitivity to chemotherapeutic agents.

To further elucidate the relationship between pyroptosis-risk scores and chemotherapy response, we employed the OncoPredict R package to estimate the half-maximal inhibitory concentration (IC50) values for a panel of commonly utilized chemotherapeutic and targeted agents across all patients (Figure 7). A lower IC50 value (μM) indicates greater drug sensitivity. Notably, 164 drugs exhibited statistically significant differences in IC50 values between the high- and low-risk groups (*p* < 0.01) (Supplementary Table 2).

We next evaluated these differences, Wilcoxon tests were performed to compare IC50 values (*p* < 0.001) between high- and low-risk groups for drugs with IC50 values below 1 μM. The IC50 values were transformed using a negative logarithmic scale, and the top six most significantly different drugs were visualized using a box-and-whisker plot. The findings revealed that vinblastine, docetaxel, bortezomib, daporinad, dactinomycin, antronium bromide exhibited potential therapeutic value in the high-risk pyroptosis group of endometrial cancer (Supplementary Figure 1).

### A key drug target for reversing pyroptosis via membrane remodeling: TSG101 interacts with pyroptosis-associated genes

**Figure 8:**
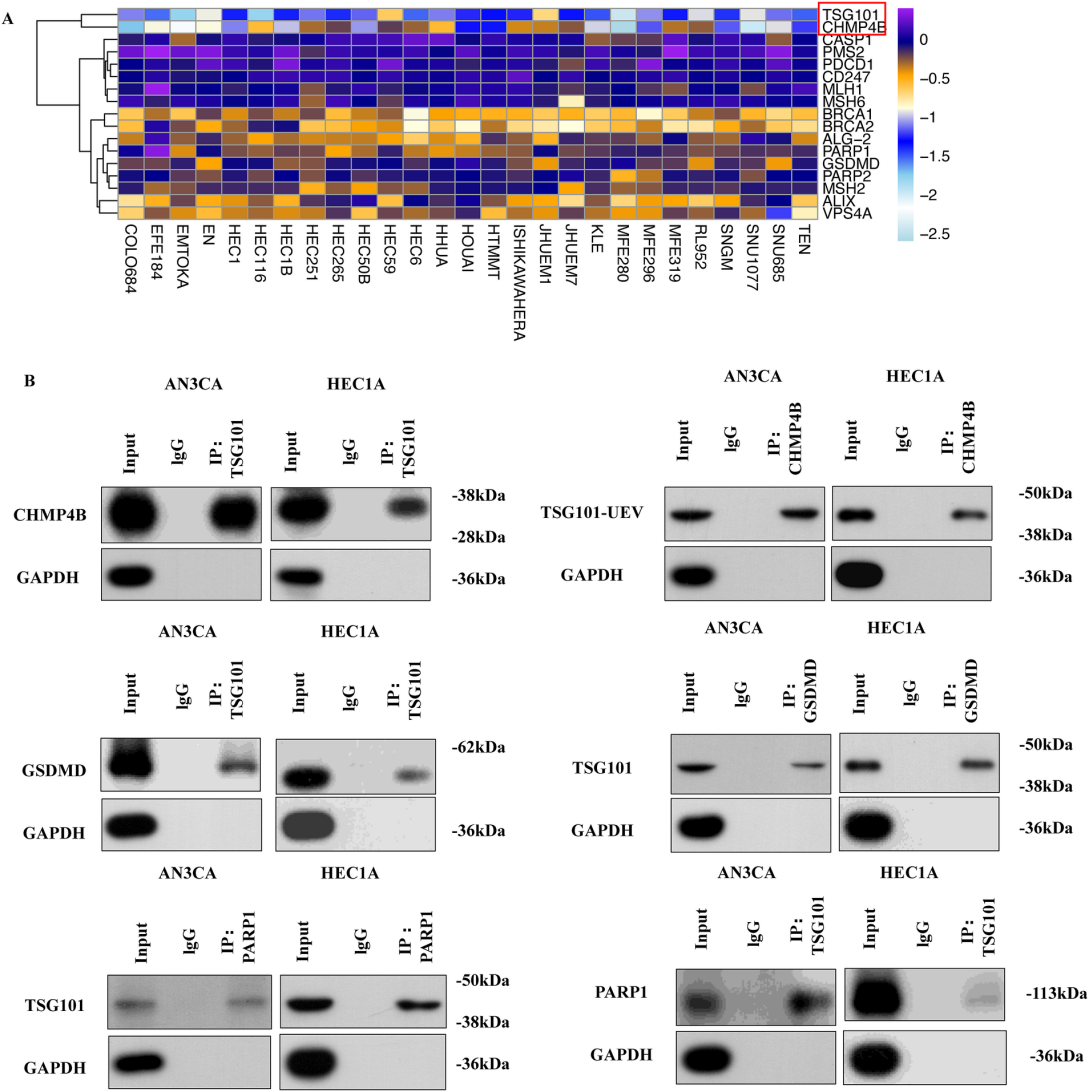
TSG101 and CHMP4B form a functional interaction network with GSDMD and PARP1 in EC. **(A)** The DepMap database identified TSG101 and CHMP4B as candidate genes with a gene effect score of less than −1 in 27 endometrial cancer (EC) cell lines. **(B)** In AN3CA and HEC1A cell lines, CO-IP was performed using His and Flag antibodies to detect the interaction between TSG101 and GSDMD, CHMP4B, and PARP1. The antibody concentration used was 1:50.

In our previous study, CHMP4B and VPS4A mitigate GSDMD-mediated pyroptosis by promoting cell membrane remodeling in endometrial carcinoma (34). To investigate the key genes involved in membrane remodeling during pyroptosis, we utilized the CRISPR-based DepMap database (35) (DepMap Public 23Q2+ Score, Chronos) to analyze the ene effect in 27 EC cell lines. Tumor susceptibility gene 101 (TSG101) and CHMP4B were identified as candidate genes with a Gene Effect score of less than −1 (Figure 8A). Furthermore, we conducted Co-IP assays in EC cell lines and confirmed pairwise interactions between TSG101 and CHMP4B, GSDMD, and poly (ADP-ribose) polymerase inhibitors (PARPi), with the TSG101 also showing association with CHMP4B (Figure 8B). Therefore, TSG101 holds promise as a key protein which regulates EC cell membrane remodeling.

**Figure 9:**
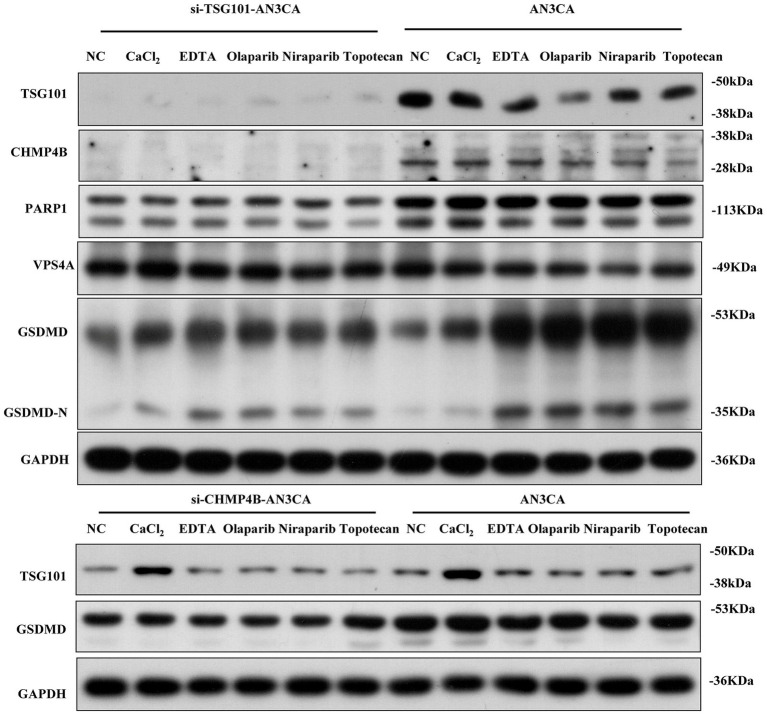
TSG101 acts upstream of CHMP4B to regulate GSDMD-mediated pyroptosis and is targeted by PARP inhibitors in endometrial cancer cells. Compared to the control group, in AN3CA cells, the addition of CaCl2, EDTA, olaparib, niraparib, and TSG101 inhibitor topotecan resulted in the downregulation of TSG101 and CHMP4B expressions, with a significant upregulation of GSDMD expression. After silencing TSG101 using siRNA, neither TSG101 nor CHMP4B expression was detected, and the expressions of PARP1, VPS4A, and GSDMD were significantly downregulated. When CHMP4B expression was silenced, TSG101 expression showed no significant change, but the expression of GSDMD was consistently downregulated across all groups. All experiments were performed in triplicate, with data presented as mean ± SD.

Recent studies show that TSG101, a differentially expressed protein in the EC pyroptosis model, may be targeted by clinically available drugs, particularly PARPi (36). To examine the upstream/downstream relationship between TSG101 and CHMP4B, as well as the effect of PARPi on TSG101 inhibition, we performed experiments in BRCA1/2 non-mutant AN3CA cells. Western blot (WB) analysis revealed that when TSG101 expression was knocked down, CHMP4B expression was absent, and the expressions of GSDMD and PARP1 were downregulated. In contrast, knocking down CHMP4B did not affect TSG101 expression, but led to a significant downregulation of GSDMD expression. These results suggest that TSG101 is upstream of CHMP4B, and CHMP4B may regulate GSDMD expression. Furthermore, after treatment with PARPi, both TSG101 and CHMP4B expressions were inhibited, while GSDMD expression was significantly upregulated (Figure 9). These findings indicate that TSG101 directly regulates CHMP4B expression, and PARPi, as an inhibitor of TSG101, downregulates CHMP4B expression and promotes GSDMD expression, potentially playing a significant role in pyroptosis, and also possesses clinically targetable drug potential.

## Discussion

Current drug therapies for endometrial cancer include (37) platinum-based chemotherapy, progestins, and targeted agents such as mTOR inhibitors and immune checkpoint inhibitors. However, due to tumor heterogeneity and therapeutic resistance (38), the clinical outcomes of advanced or recurrent endometrial cancer remain challenging. We systematically explored the role of PAGs in EC by integrating bulk RNA-seq, single-cell transcriptomics, and machine learning approaches. Our findings provide insights into the complex interplay between pyroptosis, tumor progression, and the immune microenvironment in EC. In this study, we analyzed the TCGA-UCEC cohort and identified seven representative pyroptosis-related genes in endometrial cancer (CASP9, GSDME, TIRAP, TP53, GZMB, NOD1, and GZMA) that were most effective in predicting patient survival. Our findings suggest that these genes may serve as valuable tools for guiding treatment decisions and selecting therapeutic agents.

CASP9 is an initiator caspase that plays a central role in the intrinsic (mitochondrial) apoptotic pathway. Genetic polymorphisms in the CASP9 gene have been linked to altered cancer risk across multiple tumor types (39), suggesting their potential role in modulating apoptosis and tumorigenesis. Exosome-mediated transfer of miR-769-5p from drug-resistant cells targets CASP9 and promotes the ubiquitination and degradation of p53, leading to cisplatin resistance and progression in gastric cancer (40). Overexpression of CASP9 is associated with poor overall survival (OS) in breast cancer (41). GSDME is an important member of the Gasdermin family of proteins, capable of converting caspase-3-mediated apoptosis into pyroptosis in cancer cells and activating anti-tumor immunity (42). Pyroptosis induced by GSDME promotes the release of pro-inflammatory cytokines, transforming the tumor immune microenvironment from a “cold” state to a “hot” state, significantly enhancing the effectiveness of anti-tumor immunotherapy (17). GSDME-mediated pyroptosis plays a crucial role in PD-1 blockade therapy, antibody-drug conjugate (ADC) therapy, and CAR-T therapy (43–45). However, due to the widespread expression of GSDME in nearly all body tissues and immune cells (46), it may exacerbate chemotherapy toxicity and partially hinder immune responses (47). TIRAP (Toll/interleukin-1 receptor domain-containing adaptor protein) is an adaptor molecule associated with Toll-like receptors. The T allele of the rs8177376 polymorphism in TIRAP is statistically associated with lower cervical tumor grades (48). When granzymes directly cleave and activate gasdermins to induce cell death, the expression of gasdermins in tumor cells can convert immune cell-mediated killing into inflammatory pyroptosis (43, 49), in which granzyme B cleaves gasdermin E, while granzyme A cleaves gasdermin B. As a transcription factor, TP53 directly regulates the expression of approximately 500 genes, many of which are involved in cell cycle arrest/senescence, apoptosis, or DNA damage repair—cellular responses that collectively prevent tumorigenesis (50, 51). Dysfunction of TP53 not only contributes to tumor development but also impairs the responsiveness of malignant cells to anticancer drugs, particularly those that induce DNA damage (51). NOD1 plays a dual role in cancer. It sensitizes the TNF signaling pathway to induce apoptosis and downregulate estrogen receptor expression in breast cancer (52), while also promoting cell proliferation and invasion in ovarian cancer (53), whereas its disruption in cervical and liver cancers fosters tumor progression (54, 55). In summary, pyroptosis-related genes play a critical role in tumor initiation and progression, modulation of the immune microenvironment, and the development of therapeutic resistance.

Although pyroptosis is traditionally viewed as a tumor-suppressive mechanism (56, 57), its role in cancer is complex and context-dependent. Excessive or inappropriate activation can lead to immune hyperinflammation or cytokine storms, damaging normal tissues and promoting cancer progression (58, 59). This paradox may help explain our finding that lower expression of certain PAGs is associated with longer overall survival in endometrial cancer patients. Similarly, Zhou et al. (60) identified three distinct clusters in the TCGA gastric cancer dataset and found that the cluster with the highest level of pyroptosis exhibited the longest OS. Further mechanistic studies are needed to clarify the net biological impact of pyroptosis signaling in different tumor microenvironments. In addition to prognostic modeling, we employed the oncoPredict algorithm to estimate drug sensitivity based on the gene expression profiles of EC patients. Some agents, such as vinblastine, docetaxel, bortezomib, etc., may have potential therapeutic implications for patients with elevated pyroptosis-related risk. These findings, though preliminary, provide a potential basis for exploring individualized therapeutic strategies based on PAG-driven molecular subtypes.

TSG101 is a multifunctional protein comprising a ubiquitin E2 variant (UEV) domain, a proline-rich domain (PRD), and a coiled-coil (COIL) domain. The UEV domain directly interacts with CHMP4B, facilitating its recruitment to multivesicular bodies (MVBs), which are essential for membrane remodeling (61). Inhibiting the TSG101–CHMP4B axis impairs membrane repair and compromises the survival of damaged cells (62). TSG101 also interacts with PARP1 via its COIL domain (36). PARP inhibitors (PARPi) block PARP1 activity, thereby trapping TSG101 at DNA damage sites and hindering downstream signaling (63). Additionally, due to the interaction between PARP1 and Ca^2+^, PARPi may also affect calcium homeostasis (64), functioning as dual inhibitors of TSG101 and Ca^2+^ signaling. PARPi have shown therapeutic potential in EC, even in the absence of BRCA mutations (65). These findings highlight TSG101 as a potential therapeutic target involved in membrane remodeling in EC.

Despite these promising results, several limitations must be acknowledged. First, the prognostic model was constructed and validated using TCGA data only, lacking external cohort validation. This limitation may affect the generalizability of the signature to broader EC populations. Second, while we conducted some *in vitro* assays to explore the relationships among key molecules, further biological validation at the cellular and animal levels remains essential to strengthen the causal link between PAG expression and the observed phenotypes. Third, our scRNA-seq analysis was based on a relatively small number of EC samples, which may limit the resolution and robustness of cell-type-specific expression patterns of PAGs. Future studies with larger and more diverse single-cell datasets are needed to refine these findings. Besides, artificial intelligent approaches are helpful in identifying the clinical characteristics of endometrial cancer, such as MRI radiomics effectively predicts tumor grade, myometrial invasion, LVSI, and lymph node metastasis in endometrial carcinoma, aiding diagnosis and prognosis (66). Moreover, while our study focused primarily on transcriptomic data, additional layers such as epigenetic regulation, proteomic alterations and spatial transcriptomics could further enrich our understanding of pyroptosis in EC. Lastly, although the oncoPredict-based drug sensitivity prediction offers valuable preliminary clues, actual drug responses in clinical settings may differ due to tumor heterogeneity and pharmacokinetics, which warrants further preclinical and clinical validation.

## Conclusion

In this study, we developed and validated a seven-gene pyroptosis-associated prognostic model for EC. The model demonstrated robust performance across clinical subtypes and immune phenotypes, showing strong associations with patient survival and immunotherapy response. These findings highlight the potential of PAGs as biomarkers for risk stratification and as targets for personalized treatment in EC.

## Data Availability

Publicly available datasets were analyzed in this study. These data can be found here: TCGA: https://portal.gdc.cancer.gov; GTex: https://xena.ucsc.edu; Single‐cell RNA sequencing and spatial transcriptomics data: the GEO database with accession code GSE251923.

## Supplementary material

SUPPLEMENTARY FIGURE 1 Predicting high and low risk box plots for the top six most effective chemotherapy drugs.

SUPPLEMENTARY TABLE 1 The 52 PAGs retrieved from the MSigDB database.

SUPPLEMENTARY TABLE 2 One hundred and sixty-four drugs demonstrated statistically significant differences in IC50 values between the high-risk and low-risk groups.
