# Supplementary figures and images for: Evaluation of pyroptosis-associated genes in endometrial cancer utilizing a 101-combination machine learning framework and multi-omics data
Source: Front Med (Lausanne). 2025 Jun 5;12:1590405. doi: 10.3389/fmed.2025.1590405 (PMC12176823; doi:10.3389/fmed.2025.1590405)

# Top 6 Predicted Drug Sensitivities by Risk Group

$-\log_{10}(\text{IC50})$

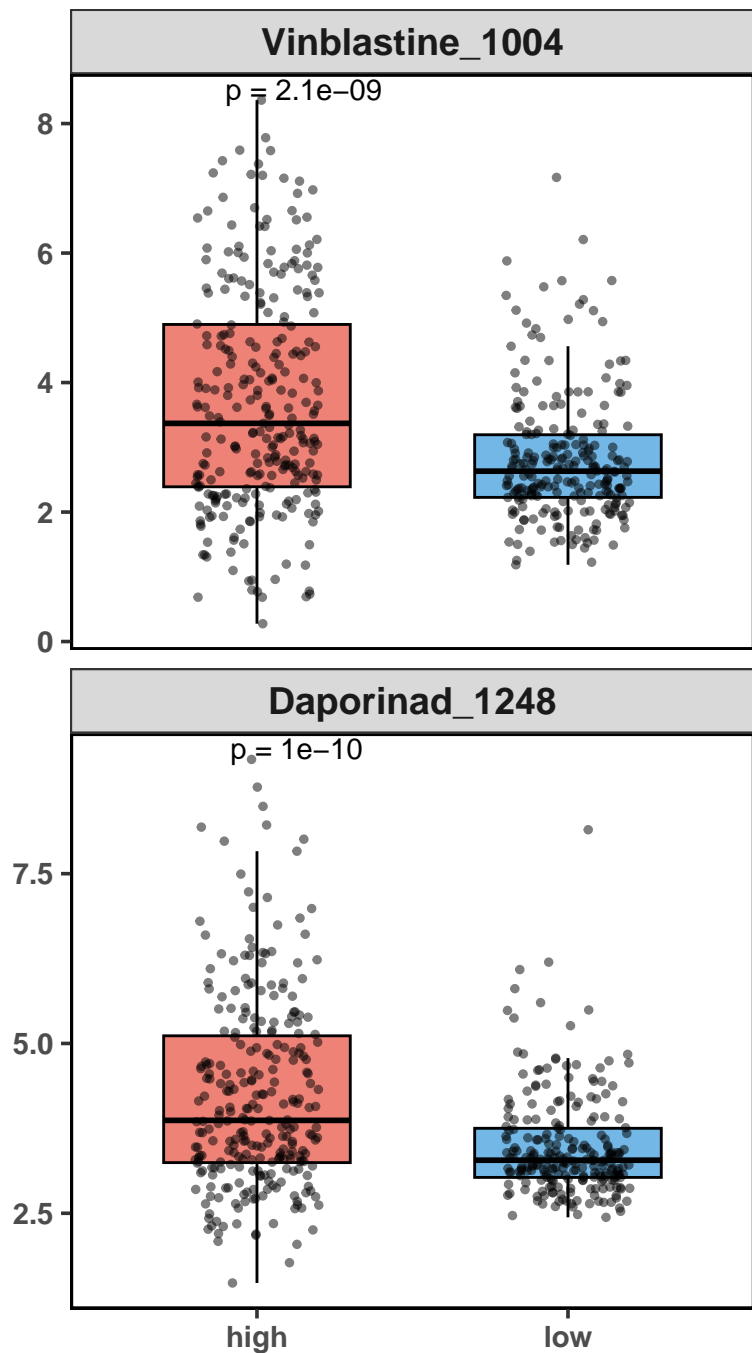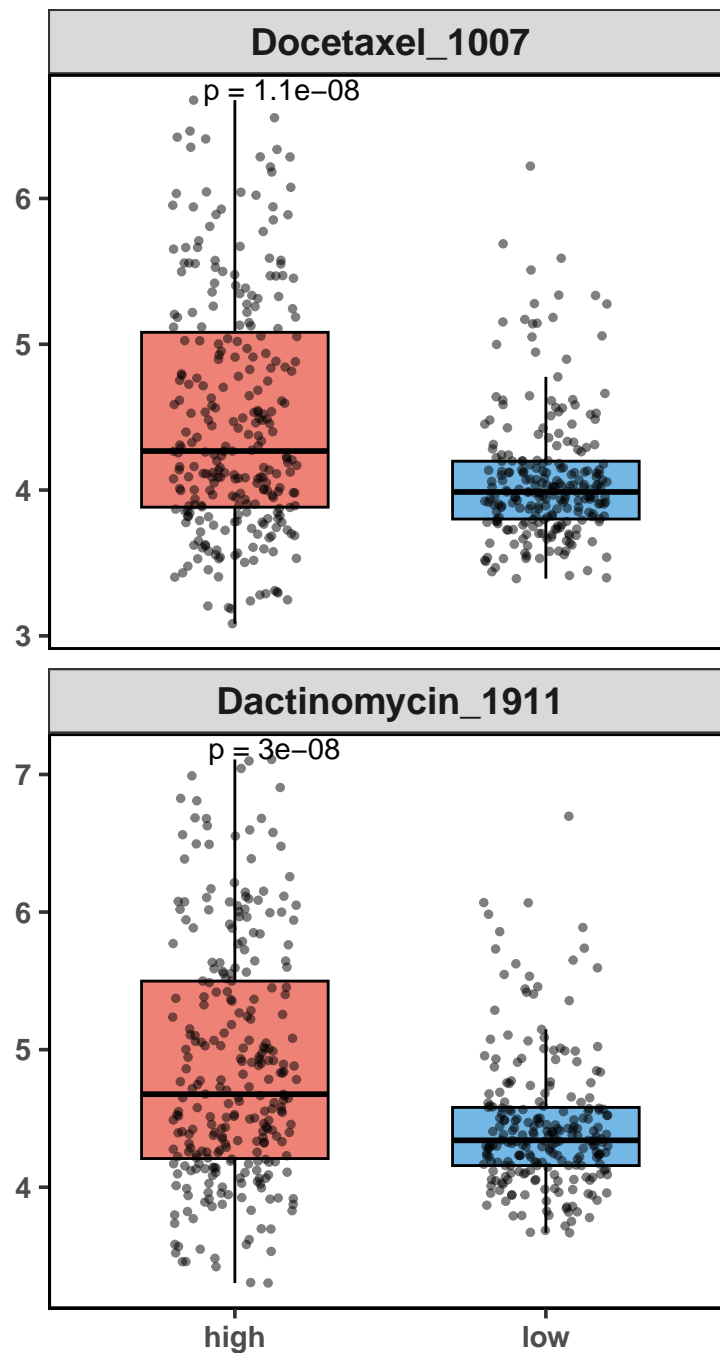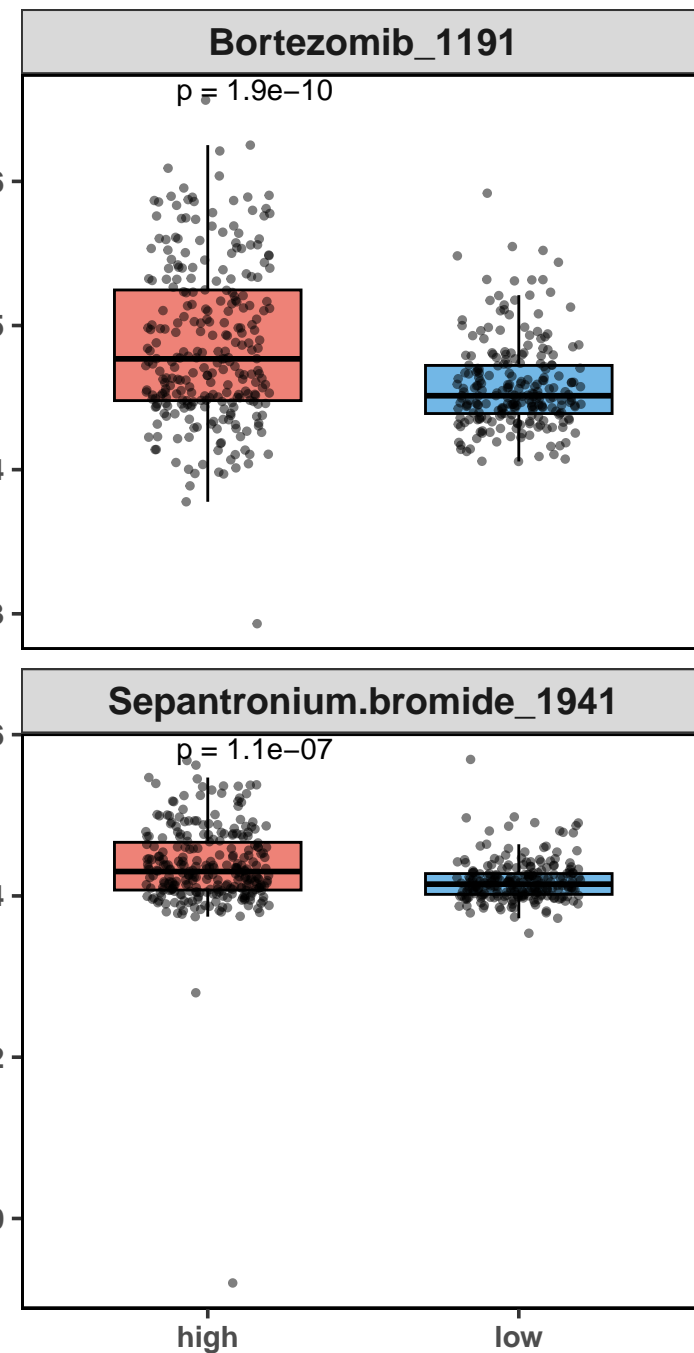

Supplement: SUPPLEMENTARY FIGURE 1 — Predicting high and low risk box plots for the top six most effective chemotherapy drugs. [file Image_1.pdf]
